# The value of dynamic contrast–enhanced MRI in characterizing complex ovarian tumors

**DOI:** 10.1186/s13048-017-0302-y

**Published:** 2017-01-14

**Authors:** Hai-Ming Li, Jin-Wei Qiang, Feng-Hua Ma, Shu-Hui Zhao

**Affiliations:** 1 Department of Radiology, Jinshan Hospital, Shanghai Medical College, Fudan University, 1508 Longhang Road, Shanghai, 201508 China; 2 Department of Radiology, Nantong Cancer Hospital, Nantong University, Nantong, Jiangsu 226361 China; 3 Department of Radiology, Obstetrics & Gynecology Hospital, Shanghai Medical College, Fudan University, Shanghai, 200011 China; 4 Department of Radiology, Xinhua Hospital, Shanghai Jiaotong University School of Medicine, 1665 Kongjiang Road, Shanghai, 2000092 China

**Keywords:** Ovarian tumors, Dynamic contrast enhanced, Magnetic resonance imaging, Differential diagnosis

## Abstract

**Background:**

The study aimed to investigate the utility of dynamic contrast enhanced MRI (DCE-MRI) in the differentiation of malignant, borderline, and benign complex ovarian tumors.

**Methods:**

DCE-MRI data of 102 consecutive complex ovarian tumors (benign 15, borderline 16, and malignant 71), confirmed by surgery and histopathology, were analyzed retrospectively. The patterns (I, II, and III) of time-signal intensity curve (TIC) and three semi-quantitative parameters, including enhancement amplitude (EA), maximal slope (MS), and time of half rising (THR), were evaluated and compared among benign, borderline, and malignant ovarian tumors. The types of TIC were compared by Pearson Chi-square *χ* ^2^ between malignant and benign, borderline tumors. The mean values of EA, MS, and THR were compared using one-way ANOVA or nonparametric Kruskal-Wallis test.

**Results:**

Fifty-nine of 71 (83%) malignant tumors showed a type-III TIC; 9 of 16 (56%) borderline tumors showed a type-II TIC, and 10 of 15 (67%) benign tumors showed a type-II TIC, with a statistically significant difference between malignant and benign tumors (*P* < 0.001) and between malignant and borderline tumors (*P* < 0.001). MS was significantly higher in malignant tumors than in benign tumors and in borderline than in benign tumors (*P* < 0.001, *P* = 0.013, respectively). THR was significantly lower in malignant tumors than in benign tumors and in borderline than in benign tumors (*P* < 0.001, *P* = 0.007, respectively). There was no statistically significant difference between malignant and borderline tumors in MS and THR (*P* = 0.19, 0.153) or among malignant, borderline, and benign tumors in EA (all *P* > 0.05).

**Conclusions:**

DCE-MRI is helpful for characterizing complex ovarian tumors; however, semi-quantitative parameters perform poorly when distinguishing malignant from borderline tumors.

## Background

Ovarian tumors represent a remarkably heterogeneous group of benign, borderline, and malignant neoplasms that usually result in significant diagnostic challenges to radiologists, surgeons, and pathologists [1–3]. The preoperative qualitative diagnosis and accurate characterization of ovarian tumors are the key steps for an optimal clinical treatment strategy, which is also helpful for improving patient prognosis [2–4].

**Fig. 1:**
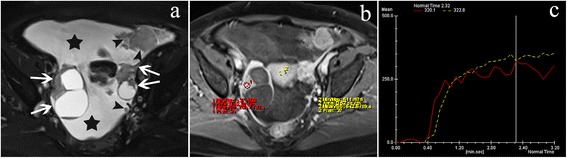
A 60-year-old woman with ovarian high-grade serous adenocarcinoma. Axial T2WI with fat suppression (**a**) demonstrated bilateral ovarian masses (*white arrow*) with peritoneal seeds (*black arrowheads*) and a large volume of ascites (*asterisk*). The mural nodule (*red ROI*) showed obvious contrast enhancement compared with the myometrium (*yellow ROI*) on contrast-enhanced T1WI with fat suppression (**b**). The mural nodule showed a curve of type III (a rapid rising and plateau pattern) (**c**)

**Fig. 2:**
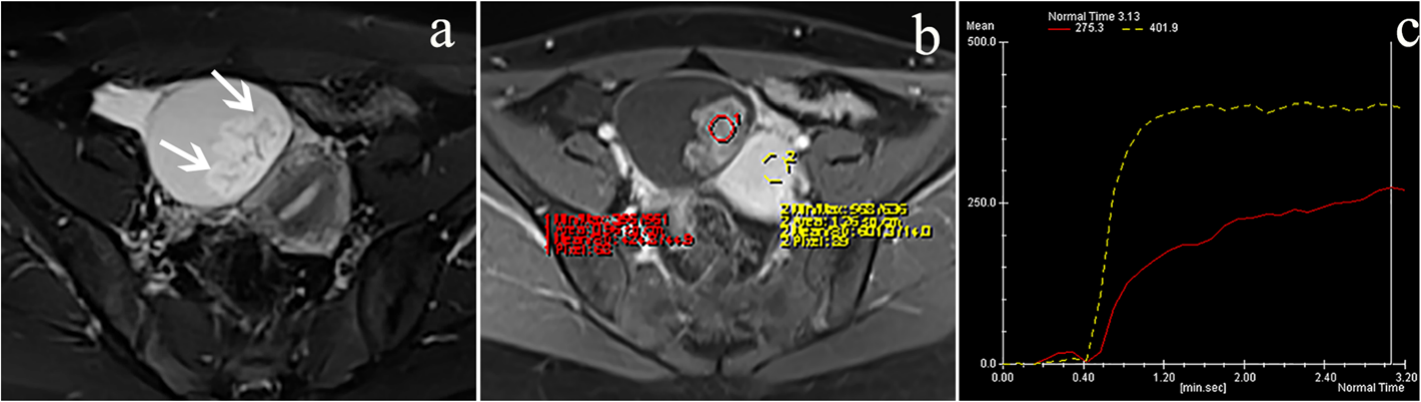
A 26-year-old woman with ovarian seromucinous borderline tumor. Axial T2WI with fat suppression (**a**) demonstrated a left-side ovarian mass with mural nodule (*white arrow*). The mural nodule (*red ROI*) showed a moderate contrast enhancement compared with the myometrium (*yellow ROI*) on contrast-enhanced T1WI with fat suppression (**b**). The mural nodule showed a curve of type II (a moderate rising pattern) (**c**)

**Fig. 3:**
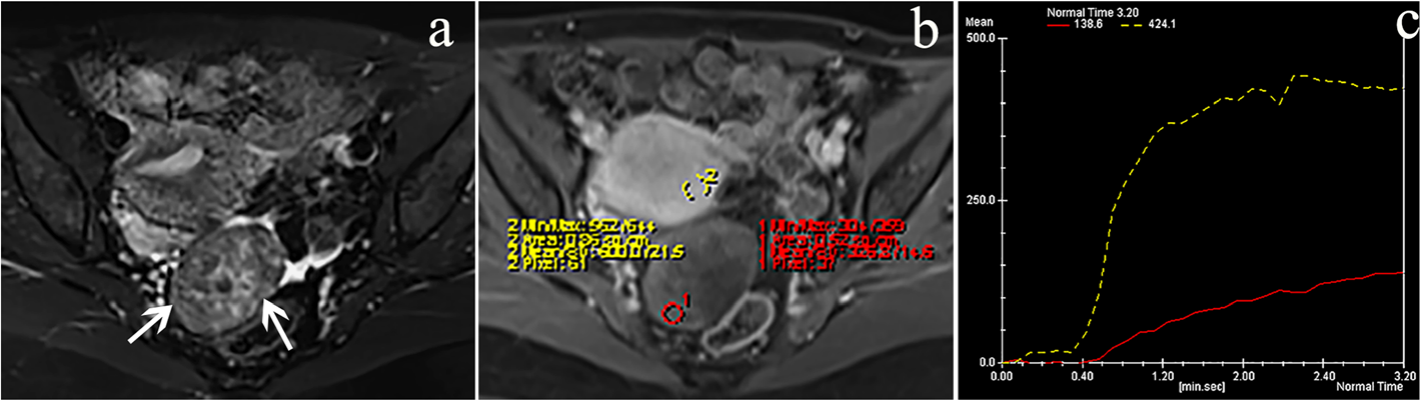
A 42-year-old woman with ovarian fibrothecoma. Axial T2WI with fat suppression (**a**) demonstrated a left-side ovarian solid mass (*white arrow*). The mass (*red ROI*) showed a mild contrast enhancement compared with the myometrium (*yellow ROI*) on contrast-enhanced T1WI with fat suppression (**b**). The mass showed a curve of type I (a gradual slow-rising pattern) (**c**)

During the past decade, some have already voiced strong concerns about the individualized treatment of ovarian tumors, especially in borderline and some subtypes of early-stage malignant tumors, because a conservative, fertility-sparing surgery can be considered for patients who wish it [5–8]. Therefore, considerable new insight has been achieved in understanding those tumors.

Ovarian tumors are mainly diagnosed by preoperative imaging. Ultrasound is generally used as a first-choice examination for screening ovarian lesions; however, it is not always helpful for the definitive diagnosis of some indeterminate ovarian tumors. Magnetic resonance imaging (MRI) has been proven to provide more anatomical information of ovarian masses and adjacent structures through its high spatial resolution and soft-tissue contrast and is helpful for differentiating diagnosis [2, 3]. However, although some morphological characteristics have been described, there are still some overlapping features on conventional MRI among ovarian tumors. As an advanced technique, dynamic contrast enhanced MRI (DCE-MRI) could be used not only to assess the microcirculatory perfusion and vascular permeability in ovarian tumors noninvasively, but provide a more comprehensive discrimination of ovarian tumors according to the time-intensity curves (TIC) and some semi-quantitative parameters [9, 10].

Previous studies have shown the advantages and the availability of semi-quantitative DCE-MRI in characterizing ovarian tumors [9–14]. The latest study including all histopathological types of ovarian masses showed that semi-quantitative DCE-MRI could discriminate malignant from borderline and benign tumors [14]. The studies that include only epithelial ovarian tumors showed similar results [9, 10]. However, standardization of scanning techniques and data processing is still needed to validate the semi-quantitative DCE-MRI in the characterization of complex ovarian masses.

## Methods

### Patient population

The institutional review board approved this retrospective study, and informed consent was waived. From February 2011 to October 2014, a total of 131 consecutive patients who were suspected of having complex ovarian tumors underwent DCE-MRI scanning. We excluded 15 patients who had not undergone surgery, 5 patients who received prior chemotherapy before MRI scanning, 4 patients with a poor imaging quality, and 5 patients without obvious solid components. Finally, 102 patients with 15 benign, 16 borderline, and 71 malignant ovarian tumors were included. All tumors were proven by surgery and histopathology within 2 weeks after the MR scan. The histopathological types of ovarian tumors are listed in Table 1. Patients’ ages ranged from 24 to 88 years (mean, 57 ± 15 years; median age, 57 years) in benign tumors, 16 to 58 years (mean, 37 ± 13 years; median age, 37 years) in borderline tumors, and 15 to 83 years (mean, 54 ± 12 years; median, 56 years) in malignant tumors, with a statistically significant difference between borderline and benign (*P* = 0.001) and between borderline and malignant tumors (*P* < 0.001).

**Table 1:** Histopathologic type in 102 ovarian tumors

Groups	Histopathologic types	Number
Benign (*n* = 15)	Fibrothecoma/thecoma	11
	Fibroma	3
	Stromal tumor with minor sex-cord elements	1
Borderline (*n* = 16)	Serous tumor	8
	Mucinous tumor	4
	Seromucinous tumor	3
	Endometrioid tumor	1
Malignant (*n* = 71)	High-grade serous adenocarcinoma	31
	Low-grade serous adenocarcinoma	3
	Endometrioid adenocarcinoma	4
	Clear-cell carcinoma	4
	Mucinous adenocarcinoma	3
	Complex adenocarcinoma	6
	Low-differentiation adenocarcinoma	5
	Sertoli-Leydig cell tumor	1
	Malignant germ-cell tumor	4
	Secondary tumor	9
	Neuroendocrine carcinoma	1

### MRI scanning

MRI was performed using a 1.5-T scanner (Avanto or Espree; Siemens, Erlangen, Germany) with a phased-array abdominal coil. The patients lay in a supine position and breathed freely during acquisition. The parameters of conventional sequences are listed in Table 2. After completing four phases of scans, the axial DCE flashes 2D, T1-weighted imaging (T1WI) with fat saturation focusing on the solid components (solid portions, vegetations, thickened septa) was performed after the intravenous administration of 0.2 mmol/kg Gadopentetate dimeglumine (Gd-DTPA, Magnevist; Bayer Schering, Berlin, Germany) at a rate of 3.0 ml/s, followed by injection of 20 ml of normal saline to flush the tube. The scanning parameters were as follows: TR/TE = 5.6/2.38 ms, slice thickness 4 mm; gap 1.0 mm; matrix 256 × 256; field of view 280–340 mm, flip angle 10°, NEX = 1. A total of 30 phases of scans were obtained sequentially at 7-s intervals for 3 min 20 s. Every phase consists of 20 images.

**Table 2:** Parameters for MRI imaging sequences

	T1WI	T2WI			Contrast-enhanced T1WI	
Parameters	Axial	Axial	Sagittal	Coronal	Sagittal	Axial
Sequence	2D SE	2D TSE	2D FSE	2D FSE	2D FSE	2D SPGR
Repetition time (msec)	761	8000	4490	4420	724	4.89
Echo time (msec)	10	83	83	83	10	2.38
Field of view (mm)	340–420	340–420	260–340	380–420	260–340	340–420
Matrix	480 × 640	256 × 256	256 × 256	320 × 320	256 × 256	256 × 256
Flip angle (degree)	150	150	144	144	150	10
Slice thickness/gap (mm)	5/1.0	5/2.0	5/1.5	5/1.5	5/1.5	5/1.5
Averages (NEX)	1	1	1	1	1	2
Acquisition time (sec)	100	100	162	96	120	42

### Semi-quantitative DCE-MRI analysis

MR images were reviewed independently by two radiologists (H.M.L. and J.W.Q., with 7 and 31 years of experience in gynecological imaging, respectively) who were blinded to the original reports (radiology, clinic, and histopathology). Any discrepancies were resolved by consensus. The MR features (tumor maximal diameters, bilaterality, shape, boundary, mass configuration, signal intensity [SI] on T2-weighted imaging (T2WI), ascites/pelvic fluid, and peritoneal implants/lymph nodes) were assessed. The time-signal intensity curve (TIC) was generated using the Mean Curve software package (Siemens). The semi-quantitative DCE-MRI parameters, including enhancement amplitude (EA), maximal slope (MS), and time of half rising (THR), were calculated. A round region of interest (ROI) of at least 1 cm^2^ was placed at targeted areas referring to T2WI and contrast-enhanced images and avoiding areas such as hemorrhage, necrosis, and major vascular structures. TIC was classified into three types referring to study of Thomassin-Naggara et al. Type I curve showed a gradual slow rising; type III had a rapid rising followed by a plateau or slow-out curve. Type II curve had a moderate rising (lower than the type III and higher than the type I). The EA is the maximal value on the *y*-axis, MS is the maximal ratio of signal intensity increasing in one scanning period, and THR is the time from the injection of Gd-DTPA to reach half of the EA. In patients with bilateral masses (25 patients with ovarian malignant tumors, 6 patients with serous borderline ovarian tumors), only the most complex mass was evaluated to reduce intra-individual influence.

### Statistical analysis

Statistical analysis was performed with SPSS 23.0 for Windows (SPSS Inc., Chicago, IL, USA). The differences in laterality, shape, boundary, mass configuration, SI on T2WI, ascites/pelvic fluid, peritoneal implants/lymph nodes, and TIC types among benign, borderline, and malignant tumors were compared using the Pearson Chi-square test or Fisher’s exact test. The differences in the age, diameters, and the parameters of EA, MS, and THR among the three groups of tumors were compared using one-way analysis of variance (ANOVA) or the nonparametric Kruskal-Wallis test. Receiver operating characteristic (ROC) curves were used to determine the cutoff values of semi-quantitative DCE-MRI parameters (MS and THR) for distinguishing between benign and malignant (borderline and malignant) tumors. A *p*-value less than 0.05 was considered statistically significant, and when the two-two comparisons were done by Pearson Chi-square test, a *p*-value less than 0.017 was considered statistically significant.

## Results

### Conventional MRI features

The MRI morphological features are summarized in Table 3. A significant difference was found in the bilaterality (*P* = 0.021, 0.006), shape (*P* = 0.001, 0.001), mass configuration (*P* = 0.009, 0.001), and peritoneal implants/lymph nodes (*P* = 0.006, 0.003) among the three groups and between benign and malignant tumours, respectively. A significant difference was also found in mass configuration between benign and borderline tumors (*P* = 0.003). However, there was no statistically significant difference between borderline and malignant tumors.

**Table 3:** Conventional MR features of ovarian tumors

MR features	Benign (*n* = 15)	borderline (*n* = 16)	malignant (*n* = 71)	*P*
Maximal diameters (cm)	8.8 ± 3.9	10.1 ± 5.0	9.9 ± 4.0	0.531^*a*^
Bilateral	0 (0)	6 (38%)	25 (35%)	0.021^*b*^
Shape				
Round/oval	13 (87%)	11 (69%)	28 (39%)	0.001^*b*^
Lobulated/irregular	2 (13%)	5 (31%)	43 (61%)	
Boundary				
Clear	15 (100%)	13 (81%)	38 (54%)	0.001^*b*^
Obscure	0 (0)	3 (19%)	33 (46%)	
Mass configuration				
Mainly cystic	0 (0)	9 (56%)	25 (35%)	0.009^*b*^
Mixed cystic and solid	2 (13%)	1 (6%)	13 (18%)	
Mainly solid	13 (87%)	6 (38%)	33 (47%)	
SI on T2-weighted imaging				
Hyperintensity	10 (67%)	13 (81%)	59 (83%)	0.345^*b*^
Iso-/hypointensity	5 (33%)	3 (19%)	12 (17%)	
Ascites/pelvic fluid	10 (67%)	8 (50%)	42 (59%)	0.638^*b*^
Peritoneal implants/lymph nodes	0 (0)	3 (19%)	28 (39%)	0.006^*b*^

Notes: ^a^ANOVA; ^b^Pearson Chi-square *χ* ^2^ test; *SI* signal intensity

**Table 4:** Comparison of semi-quantitative DCE-MRI parameters for three groups

Tumor type	Semi-quantitative DCE-MRI parameters		
	EA^*a*^	MS^*b*^	THR^*a*^
Benign	220.2 ± 90.5	6.1 ± 4.7^cd^	55.5 ± 15.4^cd^
Borderline	269.3 ± 70.9	8.5 ± 3.4	37.3 ± 15
Malignant	267.4 ± 86.2	11.0 ± 6.3	32.4 ± 8.5

Note: ^*a*^ANOVA; ^*b*^Kruskal-Wallis nonparametric test. *P* <0.05: Benign vs. Borderline (^c^); Benign vs. Malignant (^d^); *EA* enhancement amplitude, *MS* maximal slope, *THR* time of half rising

### Type of TIC

There were 59 (83%) type III, 12 (17%) type II, and no type I of TIC in malignant ovarian tumors; 4 (25%) type III, 9 (56%) type II, and 3 (19%) type I of TIC in borderline tumors; and 5 (33%) type I, 10 (67%) type II, and no type III of TIC in benign tumors, with a statistically significant difference between malignant and benign tumors (*P* < 0.001) and between malignant and borderline tumors (*P* < 0.001) (Figs. 1, 2 and 3). There was no statistically significant difference between borderline and benign tumors (*P* = 0.104). If type III TIC indicated a malignant tumor, type II and I TIC represented a benign or borderline tumor; it would generate a sensitivity of 83% (59/71), a specificity of 100% (15/15), and an accuracy of 86% (74/86) between malignant and benign tumors and a sensitivity, specificity, and accuracy of 83% (59/71), 75% (12/16), and 82% (71/87) between malignant and borderline tumors, respectively.

### Semi-quantitative DCE-MRI

The mean values of EA, MS, and THR in the three groups of tumors are shown in Table 4. In benign, borderline, and malignant tumors, the mean value of EA was 220.2 ± 90.5, 269.3 ± 70.9, and 267.4 ± 86.2, respectively, without significant difference in overall and in two-two comparisons (all *P* > 0.05). The mean value of MS was higher in malignant than in borderline tumors and higher in borderline tumors than in benign tumors, whereas the mean value of THR in malignant tumors was lower than in borderline tumors and significantly lower in borderline tumors than in benign tumors. There was a significant difference overall (*P* = 0.001, < 0.001, respectively) between benign and malignant (*P* < 0,001, 0.001, respectively), borderline tumors (*P* = 0.013, 0.007, respectively). However, no significant difference was observed between borderline and malignant tumors (*P* = 0.19, 0.53, respectively).

### Diagnostic performance of semi-quantitative DCE-MRI parameters

Receiver operating characteristic (ROC) analysis was performed for the two statistically significant parameters of MS and THR to evaluate diagnostic ability in distinguishing malignant from benign tumors and borderline from benign tumors. The area under the curve (AUC), the cutoff value, and the diagnostic performance are listed in Table 5.

**Table 5:** Receiver operating characteristic analysis of MS and THR

Parameters	AUC^a^	Threshold	Sensitivity	Specificity	Accuracy
		Borderline vs. Benign			
MS	0.763 (0.574,0.951)	≥5.2	94%	67%	81%
THR	0.829 (0.669,0.989)	≤45	88%	80%	84%
		Malignant vs. benign			
MS	0.8 (0.651,0.948)	≥6.6	77%	80%	78%
THR	0.894 (0.782,1.0)	≤45	94%	80%	92%

Note: *AUC* Area under the curve; ^a^Data in parentheses are 95% CIs

## Discussion

Angiogenesis is the process of formation of new blood vessels from preexisting blood vessels, which is the fundament of the growth, progression, invasion, and metastasis of nearly all tumors [15]. The process of angiogenesis is regulated by a variety of tumor angiogenic factors such as vascular endothelial growth factor (VEGF) and its receptor [16]. Malignant tumors were generally hypervascular with immature and fragile tumor vessels, which could increase the vascular permeability, whereas hypovascularity was characteristic of benign tumors with integrally morphological and functional vessels. DCE-MRI could make qualitative, semi-quantitative, and quantitative evaluation of blood perfusion in ovarian tumors based on the different enhancement pattern [17]. The semi-quantitative parameters were obtained by way of analyzing the patterns of TIC and were simple and feasible, whereas the quantitative parameters were derived with a complicated pharmacokinetic model.

Analyzing the type of TIC was already widely used in some clinical settings, particularly in differentiating malignant from benign tumors in breast and prostate carcinoma [18–20]. However, comprehensive use in ovarian tumors was nevertheless rare [21]. Our findings demonstrated that the TIC type was accurate for distinguishing malignant from benign ovarian tumors; however, it had a substantial overlap between borderline and malignant tumors and showed no significant difference between benign and borderline tumors. As we know, the degree of signal enhancement depends on physiological and physical factors, including tissue perfusion, arterial input function (AIF), capillary surface area, permeability, and extracellular extravascular space [22]. Angiogenesis may differ among different tumor types. Some malignant tumors, such as mucinous adenocarcinoma, may appear to be hypovascular, whereas some benign tumors such as thecoma or sclerosing stromal tumor, show abundant vascularity [2, 11]. In addition, the TIC type of tumor depends on visual and subjective assessment of contrast enhancement [20]. Therefore, TIC type alone could not definitely discriminate borderline from benign and malignant ovarian tumors. A combination of the semi-quantitative parameter analysis of TIC is needed to improve the diagnostic accuracy.

The value of EA represented the amount of contrast agent in the tumor vessels. In theory, the EA was higher in malignant than in borderline tumors and higher in borderline tumors than in benign tumors, which was confirmed by several studies [9, 10, 13, 14]. However, our study showed that the mean EA of malignant tumors was slightly lower than in borderline tumors and higher than in benign tumors (267.4 ± 86.2 vs. 269.3 ± 70.0 and 220.2 ± 90.5) (*P* > 0.05), which was not in accordance with previous studies [9, 10, 13, 14]. Therefore, further work needs to be done to verify our present findings. The MS and THR represented the rate of contrast agent in and out of the tumor vessel. Our preliminary findings showed the MS and THR were helpful for distinguishing malignant from benign tumors and borderline from benign tumors. The results of the ROC analysis were also promising, and THR was a better indicator than MS in distinguishing malignant from benign tumors (sensitivity, 94%; specificity, 80%; accuracy, 92%) and borderline from benign tumors (sensitivity, 88%; specificity, 80%; accuracy, 84%). These differences in THR and MS among the three groups of tumors were mainly due to a number of variables in angiogenesis. Malignant tumors have aberrant vascular structure, altered endothelial-cell–pericyte interactions, increased permeability, and delayed maturation of vessels, which could make the contrast agent penetrate the interstitial space more quickly [23], whereas benign tumors have fewer and relatively mature vessels, which show no obvious change in permeability and microcirculation.

Borderline ovarian tumors represented a heterogeneous group of masses with relatively good prognosis and a younger age. A conservative, fertility-sparing surgery could be considered for patients who wish to preserve fertility [5, 6]. Preoperative imaging, then, can significantly contribute by accurately identifying borderline ovarian tumors, although Medeiros’s studies and our present study showed the difficulty of conventional MRI in differentiating between borderline and malignant tumors [24]. TIC type showed a significant difference between malignant and borderline tumors, with a sensitivity, specificity, and accuracy of 83%, 75%, and 82%, respectively. Unfortunately, the three semi-quantitative parameters performed poorly and did not contribute to a differentiation of these two groups, which was inconsistent with some previous studies [9, 10, 13, 14]. The results were probably due to the diversity of samples and histopathological complexity of the tumors. For instance, mucinous borderline tumors have a lower EA and MS values and a higher THR value than other types of borderline tumors (195.9 ± 58.9 vs. 293.8 ± 57.3; 7.1 ± 5.9 vs. 8.9 ± 2.3; 42.3 ± 28.1 vs. 35.7 ± 8.8), respectively (*P* = 0.011, 0.586, 0.674). Only 4 (25%) mucinous borderline tumors were included in our study, versus 46%–50% of tumors in the studies of Thomassin-Naggara and Mansour [9, 14].

Several limitations should be addressed in our study. First, patient selection bias existed because of the retrospective nature. Second, a limited number of patients with benign and borderline tumors were included; in particular, some mucinous borderline tumors with few solid components were excluded. Third, the DCE-MRI scans were obtained sequentially for a total of 3 min 20 s in our study; a longer dynamic acquisition time should be used to yield more accurate parameters.

## Conclusions

Our preliminary findings suggest that semi-quantitative DCE-MRI is useful for distinguishing malignant from benign tumors and borderline from benign tumors, but not for differentiating malignant from borderline tumors, although there is a different TIC type. Therefore, further studies, combining other imaging techniques such as quantitative DCE-MRI, magnetic resonance spectroscopy, and diffusion-weighted imaging, are warranted.

## Abbreviations

EA: Enhancement amplitude
MS: Maximal slope
THR: Time of half rising
